# Symmetric response to competition in binary mixtures of cultivars associates with genetic gain in wheat yield

**DOI:** 10.1111/eva.13265

**Published:** 2021-07-27

**Authors:** C. Mariano Cossani, Victor O. Sadras

**Affiliations:** ^1^ South Australian Research Institute, and School of Agriculture, Food and Wine The University of Adelaide Urrbrae SA Australia

**Keywords:** competition, nitrogen, phenotype, radiation, water

## Abstract

The evolution in the definition of crop yield—from the ratio of seed harvested to seed sown to the contemporary measure of mass of seed per unit land area―has favoured less competitive phenotypes. Here we use binary mixtures of cultivars spanning five decades of selection for yield and agronomic adaptation to ask three questions. First, what is the degree of symmetry in the response of yield to neighbour; this is, if an older, more competitive cultivar increases yield by 10% with a less competitive neighbour in comparison to pure stands, would the newer, less competitive cultivar reduce yield by 10% when grown with older neighbour. Lack of symmetry would indicate factors other than competitive ability underly yield improvement. Second, what are the yield components underlying competitive interactions. Third, to what extent are the responses to neighbour mediated by radiation, water and nitrogen. A focus on yield components and resources can help the interpretation of shifts in the crop phenotype in response to selection for yield. The rate of genetic gain in yield over five decades was 24 kg ha^−1^ year^−1^ or 0.61% year^−1^. A strongly symmetrical yield response to neighbour indicates that yield improvement closely associates with a reduction in competitive ability. Response to neighbour was larger for grain number and biomass than for grain weight and allocation of biomass to grain. Under our experimental conditions, competition for radiation was dominant compared to competition of water and nitrogen. High‐yielding phenotypes had lower competitive ability for radiation but compensated with higher radiation use efficiency, a measure of canopy photosynthetic efficiency. Genetic and agronomic manipulation of the crop phenotype to reduce competitive ability could further improve wheat yield to meet the challenge of global food security.

## 1 INTRODUCTION

The definition of crop yield has evolved (Evans, 1993). For most of the history of agriculture, yield has been measured as the ratio of seed harvested to seed sown; for example, average small grain yield in Europe in the 1770s was between four and seven seeds per seed (Murphy & Hoffman, 1992). This definition of yield favoured competitive, tall plants with profuse branching. With increasing pressure for alternative uses of the available land, the definition of yield shifted from seeds per seed to the contemporary measure of mass of seed per unit land area (Evans, 1993). The selective pressure thus shifted to favour the communal phenotype first outlined by Donald (Donald, 1963, 1981; Donald & Hamblin, 1983) and updated with an evolutionary focus emphasizing multi‐level selection and kin selection (Denison, 2009, 2012, 2015; Murphy et al., 2017; Weiner et al., 2010). The core concept is that plant breeding is unlikely to improve traits shaped by natural selection over evolutionary time scales, such as the efficiency of photosynthetic enzymes, but unrealized opportunities may exist for the selection of traits that increase crop yield at the expense of plant fitness―plant breeding should be based on group selection.

Donald's communal ideotype features erect habit, reduced height, short and stiff straw and fewer tillers. The semi‐dwarf wheat and rice phenotypes of the Green Revolution realized many traits of Donald's communal phenotype, except for the extreme uniculm type (Fischer, 2020; Jennings & Dejesus, 1968). The negative correlation between yield and competitive ability has been demonstrated experimentally in species of contrasting physiology and morphology, including cereals, pulses and oilseed crops (Hamblin & Donald, 1974; Harlan & Martini, 1938; Lake et al., 2016; López Pereira et al., 2017; Reynolds et al., 1994; Sakai, 1955; Sukumaran et al., 2015; Suneson & Wiebe, 1942; Zhai et al., 2021).

Theory and empirical evidence for traits underlying the negative correlation between yield and competitive ability are most advanced in maize, for example, high‐yielding phenotypes maintain plant growth rate at flowering close to but above the minimum rate for suppression of ear growth (Andrade et al., 1999; Borrás & Vitantonio‐Mazzini, 2018; Borrás et al., 2007; Otegui et al., 2020). High‐yielding maize phenotypes feature more erect leaves that allow for higher stand density (Mantilla‐Perez & Salas Fernandez, 2017). The interaction between genotype and stand density is common in maize, highlighting genotype‐dependent variation in response to competition (Assefa et al., 2018; Hernández et al., 2014; Tollenaar, 1989; Zhai et al., 2021).

In high‐density sunflower stands, high‐yielding phenotypes self‐organize in a pattern where individuals bend away from neighbour and intercept more radiation in comparison with more competitive, lower‐yielding phenotypes that remain erect (López Pereira et al., 2017). In wheat adapted to winter–rainfall environments, phenotypes with reduced competitive ability and high yield feature more erect canopies with relaxed extinction of nitrogen relative to the extinction of radiation that lead to higher radiation use efficiency, and smaller root system with higher nitrogen uptake per unit root length (Aziz et al., 2017; Richards et al., 2019; Sadras & Lawson, 2013; Sadras et al., 2012). Modern UK wheat cultivars generally had fewer roots per plant than historic cultivars and landraces, and smaller root systems have been interpreted in terms of reduced below‐ground competition (Fradgley et al., 2020). Likewise, modern Chinese wheat cultivars had smaller root systems than old landraces (Fang et al., 2021).

Less attention has been paid to the connections between traits and the resources under competition. In genetically uniform wheat stands, intra‐specific competition was more intense for water and radiation and less intense for nitrogen with increasing availability of nitrogen (Sadras et al., 2019). In mixtures of cereal cultivars, the dominant and suppressed phenotypes varied with environment (Harlan & Martini, 1938).

Here we address three questions in binary mixtures of wheat cultivars spanning five decades of selection for yield and agronomic adaptation. First, what is the degree of symmetry in the response of yield to neighbour. If an older, low yielding, more competitive cultivar gains 10% yield when grown with newer neighbours in comparison with pure stands, would the newer cultivar reduce yield by 10% when grown with older neighbour. A high degree of symmetry would indicate genetic gain in yield associates with lower competitive ability. Second, what are the main yield components underlying competitive interactions, that is, biomass vs. allocation of biomass to grain, and seed number vs. seed weight. Third, to what extent are the responses to neighbour mediated by radiation, water and nitrogen. A focus on yield components and resources can help the interpretation of shifts in crop traits with breeding for yield as related to plant–plant interactions, and more stringently test the core hypothesis of symmetric yield response to neighbour.

## 2 METHOD

### 2.1 Field sites, cultivars, treatments and experimental design

Two field experiments were carried out on a calcareous loam (Calcarosol) at Roseworthy (34°32′ S–138°45′ E) and a silty loam over medium clay (Sodosol) at Riverton (34°9′ S, 138°44′ E), South Australia. Isbell (1996) describes these soils generically and Table S1 summarizes specifics for the soils in our experiments, including initial water and nitrogen content. Daily weather data were retrieved from nearby Australian Bureau of Meteorology stations (https://legacy.longpaddock.qld.gov.au/silo/). Crops were sown on 23 May 2019 at Roseworthy and on 3 June 2019 at Riverton, were fertilized with ammonium poly‐phosphate liquid at 50 L ha^−1^ (23.8% phosphorus, 15.9% nitrogen) and managed with local practice for the control of weeds, insects and pathogens.

**FIGURE 1:**
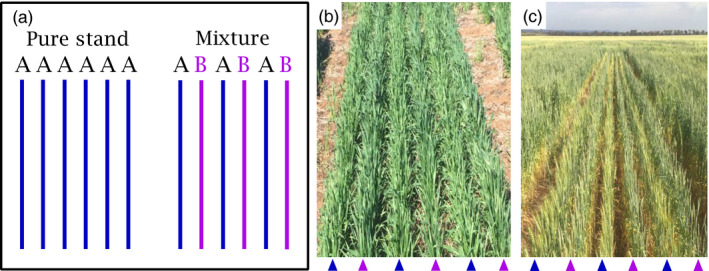
(a) Arrangement of two cultivars A and B in pure stands and alternate‐row mixtures. Illustration of crop mixtures of Mace (blue arrowhead) and Halberd (magenta arrowhead) at (b) mid‐tillering and (c) late grain filling

In each location, we established a full factorial combining (a) 16 stands, resulting from all binary mixtures of four cultivars in alternate rows and pure‐stand controls (Figure 1), (b) two stand densities d = 90 pl m^−2^ and D = 180 pl m^−2^ (c) and two rates of urea fertilizer, n = 0 kg N ha^−1^ and N = 100 kg N ha^−1^, which was split in two applications at two leaves and the beginning of stem elongation. Cultivars were Halberd (released in 1969), Spear (1984), Mace (2007) and Scepter (2015). The choice of cultivars was informed by a decade of experimentation where we phenotyped a historic collection of 13–15 cultivars for traits including yield and its components, dry matter production and partitioning, root growth, leaf photosynthesis and respiration, capture and efficiency in the use of water, nitrogen and radiation (Cossani & Sadras, 2019; Kitonyo et al., 2017; Sadras & Lawson, 2011, 2013; Sadras et al., 2012). Criteria in the selection of cultivars included similar phenology, agronomic adaptation and widespread farmer adoption (Sadras & Lawson, 2011). Our criterion of similar phenology excluded extremely late and extremely early cultivars, but a small variation in phenology was unavoidable. To account for this, crops were sampled at target phenological stages for each cultivar (Section 2.2). Individual plots were 5‐m long and included six rows separated at 0.23 m. Treatments were laid in a split–split–split block design with three replicates; locations were nested with target cultivar (main plot), neighbour (split plot), plant density (split‐split plot) and nitrogen randomized.

### 2.2 Crop development and growth

Crop phenology was monitored weekly using the Decimal Code (DC) scale of Zadoks et al. (1974) to define tillering (DC 23–24), stem elongation (DC 31), anthesis (DC 65) and physiological maturity (DC 90). Plant height from soil to the top spikelet, excluding awns, was measured with a ruler at late grain filling.

Shoot biomass was sampled when each cultivar reached tillering, 7–10 days after anthesis, and physiological maturity. All samples were taken in the two centre rows and buffers were left between samples; sample size was 0.5‐m per row at tillering and anthesis, and 1‐m per row at maturity. Shoots were oven‐dried at 65°C for 48 h to determine dry weight. Tiller fertility was calculated as the ratio of spikes to shoots in samples of 50 shoots at flowering. To separate structural and labile components of biomass, we measured the concentration of water‐soluble carbohydrates (WSC) in the anthesis sample as explained in the next section. At maturity, shoots were threshed before drying to separate grain and rest‐of‐biomass. Grain weight was determined in 300‐grain subsamples; grain number was calculated as the ratio of yield and grain weight. Reproductive allocation at maturity was calculated in two ways: as the harvest index, that is, the ratio of yield and shoot biomass, and as an allometric exponent relating grain biomass and rest‐of‐biomass in a log–log scale (Weiner et al., 2009, 2017).

### 2.3 Radiation, nitrogen and water

We measured NDVI (Greenseeker® Trimble) as a proxy for intercepted radiation (Pellegrini et al., 2020; Randall et al., 1996), the nitrogen nutrition index NNI to quantify crop nitrogen status (Gastal et al., 2015) and carbon isotope composition δ^13^C to quantify crop water status (Condon et al., 2002; Kohn, 2010; Stewart et al., 1995).

From mid‐tillering to maturity, we measured NDVI weekly, fitted polynomials to describe the time‐trajectory of NDVI and calculated the area under the curve to integrate NDVI over the season. We calculated the fraction of absorbed photosynthetically active radiation *f*APAR in pure stands using calibrations with NDVI for wheat canopies (Pellegrini et al., 2020). Cubic polynomials were fitted to describe the dynamics of *f*APAR with ontogeny; daily APAR was derived from daily *f*APAR from fitted curves and total solar radiation assuming a PAR: solar radiation ratio of 0.5 (Trápani et al., 1992). Radiation use efficiency, a measure of canopy‐level photosynthesis (Sinclair & Muchow, 1999), was calculated as the ratio between biomass at maturity and seasonal APAR (Verón et al., 2005).

The anthesis shoot samples were ground (Thomas Wiley^®^ mill model 4, Swedesboro, NJ, USA) and analysed for total nitrogen, δ^13^C, and WSC with MIR spectroscopy using a FTIR spectrometer ALPHA II (Bruker Optics Inc.). The equipment was calibrated with dry combustion for nitrogen, with a Thermo‐Finnigan Delta V Plus Isotope Ratio Mass spectrometer (IRMS, Thermo Electron) for δ^13^C and with alkaline ferricyanide decolouration method in water extracts for WSC. The NNI at anthesis was calculated as the ratio between actual and critical nitrogen concentration in shoots using the dilution curves of Hoogmoed and Sadras (2018).

### 2.4 Indices and statistical analysis

The photothermal quotient PTQ was calculated as the ratio between photosynthetically active radiation and mean temperature for the critical period between 300°Cd before and 100°Cd after anthesis using a base temperature of 4.5°C (Fischer, 1985). The rationale behind the PTQ is that grain number per m^2^, the main source of variation in yield (Sadras, 2007), is proportional to radiation driving photosynthesis, and inversely proportional to temperature driving the duration of the period of grain number determination (Fischer, 1985). Grain yield correlates with PTQ in most annual crops (Sadras & Dreccer, 2015).

The absolute (kg ha^−1^ year^−1^) and relative (% year^−1^) rates of genetic gain in yield in pure stands were calculated using the approach of Sadras and Lawson (2011). To account for variation of yield with nitrogen and stand density treatments, we (a) calculated yield deviation as the difference between the yield of a given cultivar at a given treatment and the average yield of all cultivars in each treatment and (b) calculated rates as the slope of the lest square regression between yield deviation and year of release. The relative rate was calculated in relation to the newest cultivar (Fischer, 2015). The same approach was used to calculate the rate of genetic change in other traits.

Crop traits were analysed using proc GLM with SAS for major effects and interactions (Table S2); we report *p*‐value as a continuous quantity, and Shannon information transform [*s* = −log_2_(*p*)] as a measure of the information against the tested hypothesis (Greenland, 2019). Although *s* is a function of *p*, the additional information provided is not redundant. With base‐2 log, the units for measuring this information are bits (binary digits). For example, the chance of seeing all heads in four tosses of a fair coin is 1/2^4^ = 0.0625. Thus, *p* = 0.05 represents only *s* = −log_2_(0.05) = 4.3 bits of information, ‘which is hardly more surprising than seeing all heads in four fair tosses’ (Greenland, 2019).

We defined triads of the form ‘ABA’, where *B* is the target cultivar where the trait was measured, and *A* is the neighbour (Figure 1). For each trait, we calculated response to neighbour RN (%) in relation to the corresponding monoculture (BBB):

(1) $$\mathrm{RN}\;\left(\%\right)=100\;\cdot \frac{\mathrm{ABA}}{\mathrm{BBB}}$$

We used least square linear regression to relate response to neighbour and the difference in year of release of target and neighbour; quadratic terms were tested for departures from linearity. Assuming a steady selection pressure over the period investigated (Sadras & Lawson, 2011), the rationale of our approach is that a large difference in year of release between target and neighbour, for example 46 years between Halberd and Spear, captures the putative divergence in the phenotypes.

## 3 RESULTS

### 3.1 Growing conditions

Table S1 summarizes soil conditions and Figure S1 shows the time course of weather variables during the experiment. Initial plant available water was 92 mm at Riverton and 39 mm at Roseworthy. Seasonal rainfall was 216 mm and rainfall exceeded evaporative demand during most of the season at Riverton, while seasonal rainfall was 173 mm and matched evaporative demand during most of the season at Roseworthy. The photothermal quotient during the critical period was similar in both locations: 1.21 MJ m^−2^ d^−1^°C^−1^ at Riverton and 1.26 MJ m^−2^ d^−1^°C^−1^ at Roseworthy. Mean temperature during the critical period was higher at Riverton (13.6°C) than at Roseworthy (11.7°C), causing a shortening of 13 days in the critical period at Riverton compared to Roseworthy. Two consecutive days during the critical period had maximum temperature over 30°C at Riverton; temperature over 30°C disrupts wheat reproduction (Saini & Aspinall, 1982; Saini et al., 1983).

### 3.2 Yield in pure stands increased steadily with year of cultivar release

**FIGURE 2:**
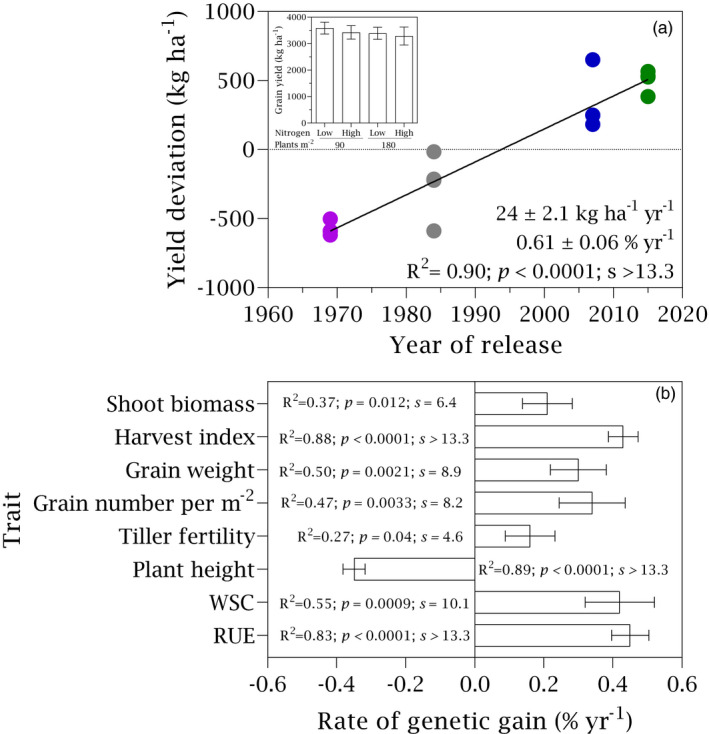
(a) Yield in pure stands as a function of year of cultivar release in crops grown under two stand densities (d = 90, D = 180 plants m^−2^) and two nitrogen rates (n = 0, N = 100 kg ha^−1^). Inset shows the yield (±SE) for each treatment averaged across varieties. Data are averaged across two locations, Roseworthy and Riverton. Solid line is the least square regression. Absolute (kg ha^−1^ year^−1^) and relative (% year^−1^) rates ±SE are shown. (b) Relative rate of genetic change in crop traits. WSC is concentration of water‐soluble carbohydrates in shoot at anthesis, and RUE is seasonal radiation use efficiency

Yield in pure stands increased linearly at 24 kg ha^−1^ year^−1^ or 0.61% year^−1^ (Figure 2a). There were not differences in the relative rate of genetic gain with stand density, nitrogen or their interaction (*p* > 0.57, *s* < 0.8). Genetic gain in yield associated with shorter plants, higher concentration of water‐soluble carbohydrates in shoot at anthesis, higher tiller fertility, more grains per m^2^, heavier grains, more biomass at maturity and higher harvest index (Figure 2b; Table S3). Selection for yield also enhanced radiation use efficiency (Figure 2b; Section 4.2).

### 3.3 Yield response to neighbour was strongly symmetric

**FIGURE 3:**
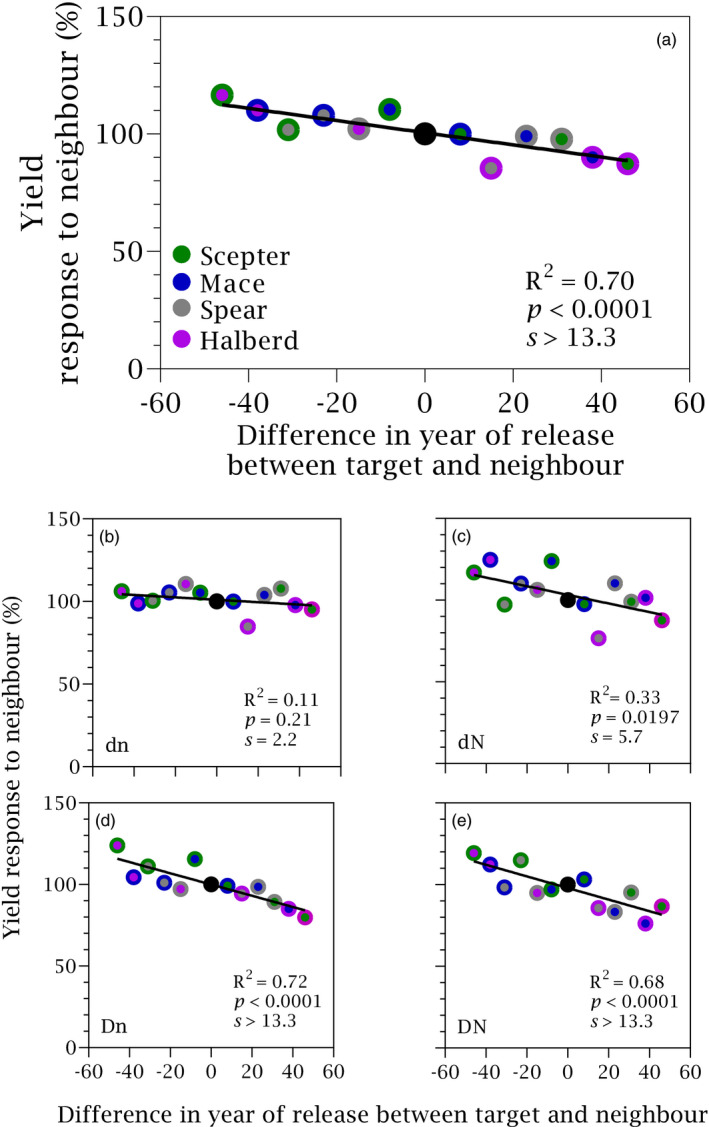
(a) Yield response to neighbour as a function of the difference in year of release between target and neighbour cultivars. Yield is averaged across two locations, two stand densities (d = 90 plants m^−2^, D = 180 plants m^−2^), and two nitrogen rates (n = 0 kg ha^−1^, N = 100 kg ha^−1^). (b–e) Yield response to neighbour as a function of the difference in year of release between target and neighbour cultivars for each density‐nitrogen combination averaged for the two locations. Symbols: filling colour shows target and edge colour shows neighbour, with black circle denoting pure stand. Solid lines are least squares regressions, with slopes shown in Table 1

Halberd, the oldest cultivar in our set, increased yield by 17 ± 0.03% with Scepter neighbour in comparison to pure Halberd stands. Scepter, the newest cultivar, decreased yield by 13 ± 0.05% with Halberd neighbour in comparison with pure Scepter stands. All 12‐pairwise combinations of cultivars grown under eight conditions aligned in a plot of yield of target cultivar relative to pure stand (Equation 1) and the difference in year of release between target and neighbour (Figure 3a). The fitted line passed through the (0, 100) coordinate (*p* < 0.0001, *s* > 13.3) supporting the conclusion of symmetry in the response of yield to neighbour. The scatter in Figure 3a was associated with stand density and nitrogen, with slopes from zero with low density and low nitrogen to −0.36% year^−1^ for high density and high nitrogen (Figure 3b–e; Table 1).

**TABLE 1:** Slope ± SE (% year^−1^) of the least square regression between trait response to neighbour and the difference in year of release between target and neighbour cultivars in binary mixtures grown at two stand densities (d = 90 plants m^−2^, D = 180 plants m^−2^) and two nitrogen rates (n = 0 kg ha^−1^, N = 100 kg ha^−1^), averaged for two locations, Roseworthy and Riverton. Rates are coloured according to the Shannon information transform (Greenland, 2019)

Trait	dn	dN	Dn	DN
Grain yield	−0.073 ± 0.056	−0.260 ± 0.10	−0.340 ± 0.057	−0.360 ± 0.065
Grain number per m^2^	0.0097 ± 0.057	−0.190 ± 0.096	−0.220 ± 0.066	−0.290 ± 0.053
Grain weight	−0.074 ± 0.022	−0.074 ± 0.036	−0.110 ± 0.038	−0.055 ± 0.053
Shoot biomass at maturity	−0.029 ± 0.050	−0.170 ± 0.089	−0.260 ± 0.046	−0.350 ± 0.039
Harvest index	−0.048 ± 0.023	−0.094 ± 0.028	−0.080 ± 0.032	−0.004 ± 0.042
Allometric exponent at maturity	−0.015 ± 0.007	−0.025 ± 0.008	−0.024 ± 0.009	−0.005 ± 0.012
Tiller fertility	−0.077 ± 0.055	−0.170 ± 0.073	−0.120 ± 0.038	−0.190 ± 0.063
Shoot biomass at anthesis	−0.240 ± 0.105	−0.029 ± 0.129	−0.30 ± 0.067	−0.250 ± 0.061
δ^13^C at anthesis	0.002 ± 0.008	0.004 ± 0.008	−0.008 ± 0.008	0.017 ± 0.014
Nitrogen Nutrition Index at anthesis	−0.190 ± 0.198	0.081 ± 0.117	−0.150 ± 0.157	−0.180 ± 0.107
WSC at anthesis	−0.259 ± 0.083	−0.549 ± 0.149	−0.204 ± 0.113	−0.515 ± 0.173
Plant height at maturity	0.009 ± 0.017	−0.035 ± 0.011	0.021 ± 0.017	−0.014 ± 0.027

Note

*S* = 0.0 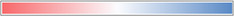 *S* = 13.3.

Owing to the linear increase of yield in pure stands with year of release (Figure 2a), the relationship between response to competition and the difference in year of release between target and neighbour (Figure 3a) implies a relationship between response to competition and yield in pure stands. However, yield response to competition is a function of yield in pure stands by definition (Equation 1). To avoid spurious correlations (Brett, 2004), we favoured difference in year of release as independent variable.

### 3.4 Biomass and grain number primarily mediated yield response to neighbour

**FIGURE 4:**
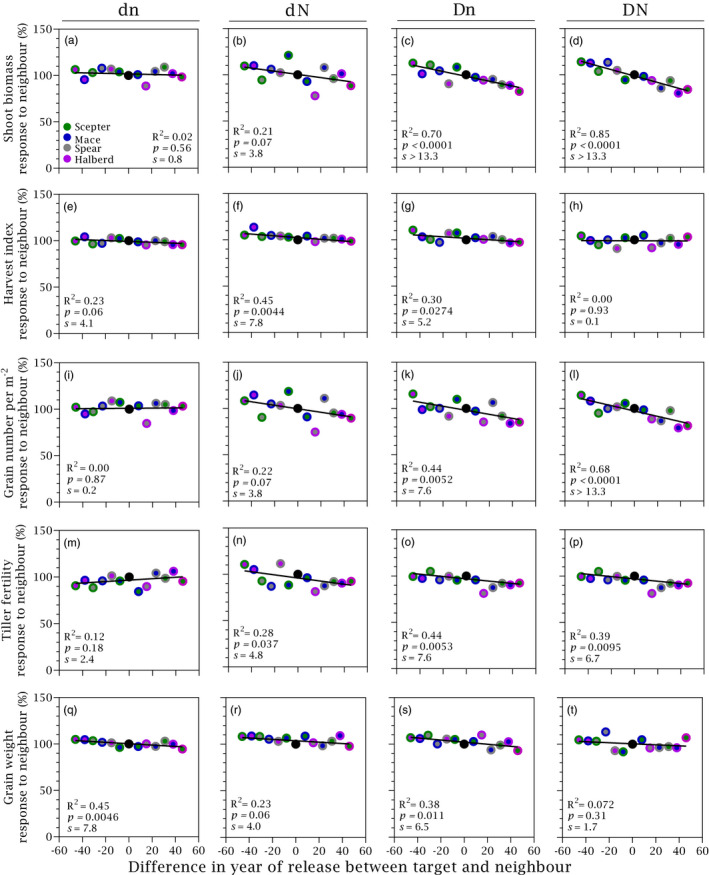
Response to neighbour of grain yield components as a function of the difference in year of release between target and neighbour cultivars. (a–d) Shoot biomass at maturity, (e–h) harvest index, (i–l) grain number per m^2^, (m–p) tiller fertility and (q–t) grain weight. Data are averaged across two locations, two stand densities (d = 90 plants m^−2^, D = 180 plants m^−2^) and two nitrogen rates (n = 0 kg ha^−1^, N = 100 kg ha^−1^). Symbols: filling colour shows target and edge colour shows neighbour, with black circle denoting pure stand. Solid lines are least squares regressions, with slopes shown in Table 1

Figure 4 and Table 1 show responses to neighbour for yield components. Maturity biomass was more responsive to neighbour than harvest index, and grain number was more responsive than grain weight. At high density and low nitrogen, the response to neighbour of maturity biomass was 3.25‐fold the response of harvest index, and the response of grain number was double than the response of grain weigh. Consistent with the response of yield, the response to neighbour of maturity biomass and grain number were stronger in high‐density stands (Figure 3b,c vs. Figure 4). For both harvest index and grain weight, response to neighbour was only apparent under low nitrogen. Allometric exponents captured nitrogen and density‐dependent neighbour effects similarly to harvest index (Table 1, Figure S2). The response of grain number to neighbour was related to tiller fertility (Figure 4m–p; Table 1).

### 3.5 Newer cultivars were shorter and intercepted less radiation but had higher radiation use efficiency than older ones

**FIGURE 5:**
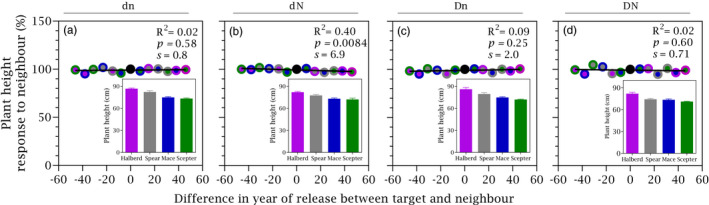
Response to neighbour of plant height as a function of the difference in year of release between target and neighbour cultivars for crops grown at two stand densities (d = 90 plants m^−2^, D = 180 plants m^−2^) and two nitrogen rates (n = 0 kg ha^−1^, N = 100 kg ha^−1^), averaged across two locations. The solid line is the least squares regression, with slopes shown in Table 1. Insets show average plant height in pure stands, with error bars showing one standard error. In both scatter plot and bar chart, magenta is Halberd, grey is Spear, blue is Mace, and green is Scepter. In scatter plots, filling colour shows target and edge colour shows neighbour, with black circle denoting pure stand

**FIGURE 6:**
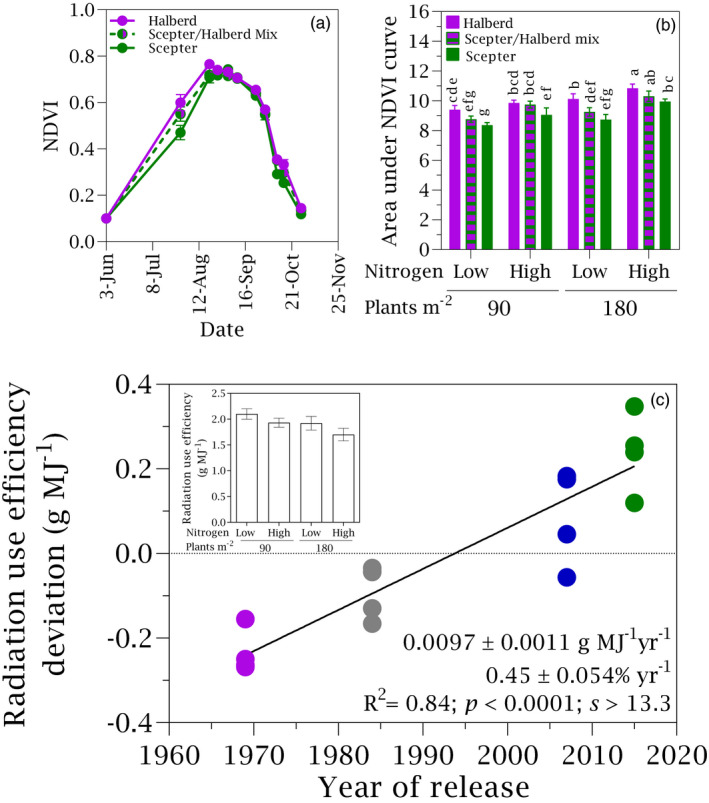
(a) Example of seasonal dynamics of NDVI for Halberd, the oldest cultivar, Scepter, the newest cultivar and their mixture. (b) Area under the NDVI curve for crops grown at two stand densities and two nitrogen rates, averaged for two locations. Letters indicate *p* < 0.05, *s* > 4.3 from ANOVA. (c) Radiation use efficiency in pure stands as a function of year of cultivar release for crops grown at two stand densities (d = 90 plants m^−2^, D = 180 plants m^−2^) and two nitrogen rates (n = 0 kg ha^−1^, N = 100 kg ha^−1^), averaged across two locations. Absolute (g MJ^−1^ year^−1^) and relative (% year^−1^) rates ±SE are slopes from least square regressions (solid line). Inset is average radiation use efficiency for the combinations of stand density and nitrogen rate. Error bars are two standard errors in both (a) and inset (c) and one standard error in (b)

Newer cultivars were shorter (inset Figure 5, Figure 2b), and plant height did not respond to neighbour, except for a slight response in high‐nitrogen, low‐density stands (Figure 5). Lodging was not apparent in our experiment, even for the oldest and tallest Halberd (Figure 1b,c). The area under the NDVI curve was higher for Halberd than for Scepter and intermediate for their mixture (Figure 6a,b). In pure stands, radiation use efficiency increased from older to newer cultivars at a rate of 0.0097 g MJ^−1^ year^−1^ or 0.44% year^−1^ (Figure 6c).

### 3.6 Plant water and nitrogen status did not respond to neighbour, and WSC responded asymmetrically

**FIGURE 7:**
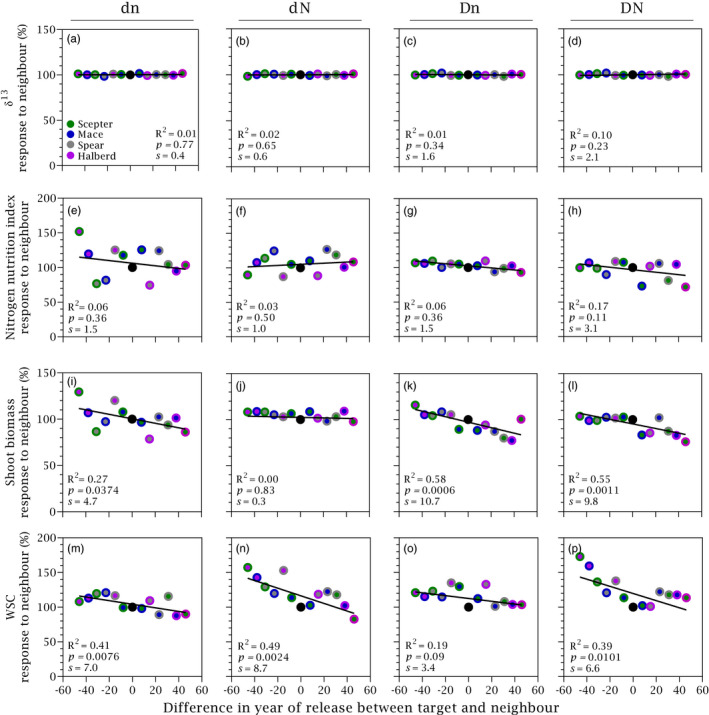
Response to neighbour of crop traits at anthesis as a function of the difference in year of release between target and neighbour for crops grown at two stand densities (d = 90 plants m^−2^, D = 180 plants m^−2^) and two nitrogen rates (n = 0 kg ha^−1^, N = 100 kg ha^−1^), averaged across two locations. (a–d) Carbon isotope composition, an integrated measure of water status, (e–h) nitrogen nutrition index, (i–l) shoot biomass at anthesis and (m–p) concentration of water‐soluble carbohydrates (WSC) in shoots. Symbols: filling colour shows target and edge colour shows neighbour, with black circle denoting pure stand. Solid lines are least squares regressions, with slopes in Table 1

Carbon isotope composition (Figure 7a–d) and the nitrogen nutrition index at anthesis (Figure 7e–h) did not respond to neighbour. Anthesis biomass increased in older cultivars with newer neighbour and decreased symmetrically in newer cultivars with older neighbour in high‐density stands irrespective of nitrogen supply (Figure 7i–l). Concentration of WSC increased in older cultivars with newer neighbour and vice‐versa in high‐nitrogen, high‐density stands, and to a lesser extent in low nitrogen, low density stands (Figure 7m–p; Table 1). The response of WSC to neighbour was asymmetrical in high nitrogen irrespective of plant density, where the increase in WSC of older cultivars with newer neighbour was larger than the reduction of newer cultivars with older neighbour (Figure 7n,p).

## 4 DISCUSSION

### 4.1 Symmetric response of yield to neighbour highlights the communal ideotype

Crop yield is a population attribute (Sadras, 2019) whereby the behaviour of the plant becomes subordinated within that of the population (Harper, 1977). The association between high grain yield per unit land area and low competitive ability led to the concept of the communal plant (Donald, 1963, 1981). Updated theory (Denison, 2009, 2012, 2015; Murphy, Swanton et al., 2017; Weiner et al., 2010) and empirical evidence (Hamblin & Donald, 1974; Harlan & Martini, 1938; Lake et al., 2016; López Pereira et al., 2017; Reynolds et al., 1994; Sukumaran et al., 2015; Suneson & Wiebe, 1942) support this proposition. Here we report a symmetric response of grain yield to neighbour providing new evidence in favour of Donald's communal plant.

Although we only tested four cultivars spanning almost 50 years of breeding, the rate of genetic gain in yield was similar to the rates derived in independent studies using larger collections of cultivars over a similar period (Cossani & Sadras, 2019; Sadras & Lawson, 2011). Our average rate of 0.61 ± 0.06% year^−1^ compares with an average genetic gain of 0.55 ± 0.04% year^−1^ for a worldwide set of 22 studies (Fischer et al., 2014). The absolute rate of genetic gain is proportional to the potential of the environment, for example, rates over 50 kg ha^−1^ year^−1^ have been reported for high‐yielding conditions of the UK and France, which are at least double the typical rates of drier environments like Australia (Sadras et al., 2016). Normalized rates of genetic gain in yield are considered independent of environmental conditions (Fischer et al., 2014). Consistently, we found similar relative rates of genetic gain across stand density and nitrogen treatments.

Lack of symmetry, that is an increase in the yield of an old cultivar with newer neighbour that does not match the reduction of yield of the newer cultivar with older neighbour would indicate that part of the genetic gain in yield is unrelated to competitive ability. Hence, the high symmetry in the response to neighbour in our study indicates a dominant role of reduced competitive ability as a driver of yield improvement. Symmetry of the response to neighbour was also apparent for yield components tiller fertility, grain number and maturity biomass (Figure 4). The symmetry in the response on tiller fertility indicates neighbour interference (sensu Harper, 1977) at early stages of crop development (Slafer et al., 2014). Early experiments reported lack of symmetry in wheat response to neighbour whereby the yield gain of a tall cultivar with short neighbour was smaller (Austin & Blackwell, 1980) or larger (Jensen & Federer, 1964) than the yield reduction of the short cultivar with a tall neighbour. Lodging is typical of older, taller cultivars with high availability of resources, but lodging was not apparent or not reported in these early studies. The response to neighbour was also asymmetrical in mixtures of Norin 12 upland rice and red rice, a primitive strain of Indica rice, whereby the reduction in yield of Norin 12 with red rice neighbour was larger than the increase in yield of red rice with the less competitive Norin 12 (Sakai, 1955). The strong symmetry in our crop mixtures is therefore not a trivial finding.

### 4.2 Older, taller cultivars outcompeted newer, shorter cultivars for radiation, but newer cultivars had higher radiation use efficiency

Under our experimental conditions, the effect of neighbour was not apparent for water and nitrogen status of plants at anthesis, irrespective of stand density and nitrogen supply (Figure 7). In drier conditions and with lower supply of nitrogen, competition for soil resources could be more relevant and needs attention. Rainfall exceeding evaporative demand during most of the pre‐flowering period (Figure S1) accounts for the lack of variation in crop water status. In this environment, the relative importance of competition for radiation, water and nitrogen changes with sowing date, with late‐May to early‐June sowings less likely to feature nitrogen deficit (Sadras et al., 2019). Competition for radiation, closely related to plant height, was therefore the main driver of neighbour effects (Figures 5 and 6). In two out of three conditions where yield responded to neighbour, carbohydrate reserves at anthesis also responded to neighbour (Figure 7n,o), reinforcing the dominant role of competition for radiation and carbon assimilation. However, the response of carbohydrate reserves to neighbour lacked symmetry—concentration of water‐soluble carbohydrates increased in Halberd with Scepter neighbour but did not reduce in the same proportion in Scepter with Halberd neighbour. The reason for this lack of symmetry is unknown; it has been speculated that high concentration of water‐soluble carbohydrates could play an osmotic role and increase in response to selection for tolerance to aphids (Sadras et al., 2020, 2021).

The superior yield of stands with shorter plants at the core of the Green Revolution was primarily attributed to higher allocation of biomass to grain and reduced lodging with high‐nitrogen fertilizer (Fischer & Quail, 1990). A complementary explanation is that shorter plants are less competitive for radiation; shading during the critical period reduces grain number and yield (Fischer, 1985). Indeed, plant height and canopy geometry are critical to competition for radiation (Austin & Blackwell, 1980; Barnes et al., 1990; Cousens et al., 2003; Ford & Diggle, 1981; Jensen & Federer, 1964, 1965). In our study, older, taller cultivars with more planophile canopies intercepted more radiation than their newer, shorter counterparts with more erect leaves (Sadras et al., 2012). Higher radiation use efficiency in the newer cultivars compensated for their lower ability to intercept radiation.

The genetic rate of increase in radiation use efficiency in our study (Figure 6c) is similar to the rates reported for a larger set of historic cultivars adapted to winter–rainfall environments of Australia and for British cultivars (Sadras et al., 2012; Shearman et al., 2005). Superior yield of erectophile phenotypes is well documented in wheat (Richards, 2000; Richards et al., 2019). Taller cultivars with denser, planophile canopies can benefit from shorter neighbour as shown in experiments using cultivar mixtures, comparisons of adjacent rows between plots, and plots with different plant height (Austin & Blackwell, 1980; Fischer, 1978; Jensen & Federer, 1964, 1965; Kawano et al., 1974; Mumaw & Weber, 1957; Puckridge & Donald, 1967; Schutz & Brim, 1967).

Reynolds et al. (1994) reduced competition for radiation in wheat stands by bending adjacent rows away from the central rows at the developmental stage of flag‐leaf ligule emergence, when interplant competition was assumed to peak. The average yield response was 25%, with higher response to competition in lines with low yield potential compared with their high yield potential counterparts. Concurrent with our findings, the authors concluded that the greater yield of high‐yielding lines was related to ‘their better adaptation to interplant competition’. They further propose that ‘genes conferring yield potential through improved adaptation to the crop environment are associated with a less competitive phenotype’.

### 4.3 Agricultural implications: genetic and agronomic approaches to reduce intra‐specific competition can improve crop production

Wheat contributes about 20% of energy and protein in human diets worldwide and will remain central to global food security in the foreseeable future (Shiferaw et al., 2013). Meeting the projected demand of staple crops by 2050 requires a minimum yield increase of 1.1% year^−1^ relative to 2010 yield (Fischer & Connor, 2018). Crop production can be increased genetically, agronomically and exploiting the synergies between breeding and agronomy (Fischer, 2009).

**FIGURE 8:**
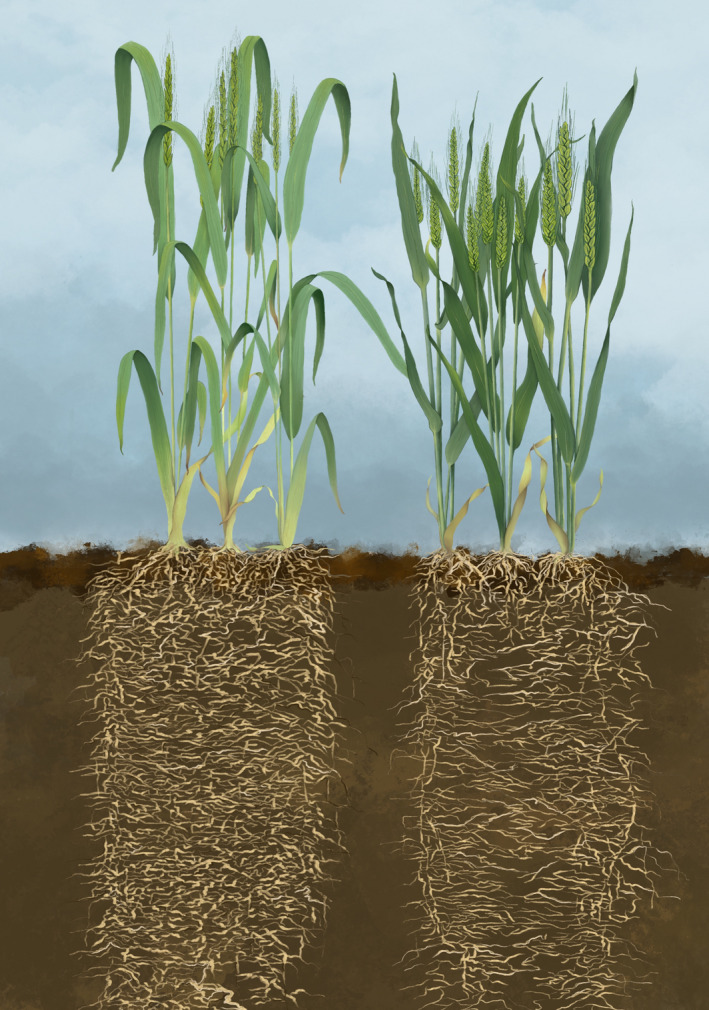
Decades of selection for yield and agronomic adaptation shifted key traits from a low yielding, more competitive phenotype (left) to a high yielding, less competitive phenotype (right). The less competitive [Figure 3, this study], higher yielding phenotype [Figure 1] is shorter [Figure 5] and intercepts less radiation [Figure 6]. Higher radiation use efficiency [Figure 6c] compensates for the lower interception of radiation in the less competitive phenotype. Higher radiation use efficiency is independent of photosynthesis and respiration at leaf level and relates to an erectophyl canopy that favours more radiation and higher nitrogen concentration in leaves at the bottom of the canopy. The less competitive phenotype has a smaller root system (Aziz et al., 2017; Fradgley et al., 2020) with compensatory higher nitrogen uptake per unit root length (Aziz et al., 2017)

The symmetry in yield response to neighbour under realistic field conditions in our study strongly supports the link between high yield and low competitive ability. Figure 8 and supporting references update Donald's communal ideotype, a benchmark for both genetic and agronomic improvement. The less competitive, higher yielding phenotype is shorter and intercepts less radiation. Higher radiation use efficiency compensates for the lower interception of radiation in the less competitive phenotype, is independent of leaf photosynthesis and respiration, and relates to an erectophyl canopy that favours more radiation and higher nitrogen concentration in leaves at the bottom of the canopy. Lower partitioning to roots may have also contributed to higher radiation use efficiency. Some historic collections of wheat revealed selection for yield favoured higher rate of light‐saturated leaf photosynthesis (e.g. Sun et al., 2014), but the link between short‐term leaf photosynthetic rate and seasonal photosynthesis of canopies has not been established in these studies; leaf photosynthesis rarely scales to canopy photosynthesis (Pettigrew et al., 1989; Sinclair et al., 2019). The less competitive phenotype has a smaller root system with compensatory higher nitrogen uptake per unit root length (Figure 8). Despite significant effort, enhancing radiation use efficiency and nitrogen uptake remain elusive (Furbank et al., 2019; Lammerts van Bueren & Struik, 2017; Sinclair et al., 2019). Selective pressure for lower competitive ability may indirectly favour canopy photosynthesis and nitrogen uptake. Lower competitive ability could be selected for phenotypically based on growth measured in centre and border rows (Sadras & Lawson, 2011), with molecular tools (Sukumaran et al., 2015), and with targeted genetic manipulation of phytochromes involved in the early‐perception of neighbour and shade‐avoidance syndrome (Boccalandro et al., 2003; Wies & Maddonni, 2020).

A corollary of the communal plant concept is that early generation selection should target traits associated with reduced competitive ability; others advocate for selection of plants with high yield under nil‐competition using a honeycomb design to control for soil variation (Fasoulas & Fasoula, 1995; Fischer, 2020). Whereas yield‐density curves may level off at low stand density in some crops, gene expression and plant phenotype depend on both stand density and genetic identity of neighbouring individuals (Bowsher et al., 2017; Crepy & Casal, 2015; Geisler et al., 2012; Murphy, Swanton et al., 2017; Murphy et al., 2017). Overlooking plant–plant relations is a major source of inefficient plant phenotyping, even under controlled conditions (Chen et al., 2019; Sadras, 2019).

Precision seeding could be used to reduce the rectangularity of crop arrangements to reduce intra‐specific competition (Barbieri et al., 2008; Fischer, 2020; Maddonni et al., 2001). The strong symmetry in the response of binary mixtures in our study is consistent with the common lack of benefit in yield of well‐protected crop mixtures (Haghshenas et al., 2021; Vidal et al., 2020). Irrespective of the source, breeding or agronomy, higher yielding, less competitive phenotypes require more stringent weed control as intra‐specific and inter‐specific competitive ability correlate (Coleman et al., 2001; Evans, 1998; McDonald & Gill, 2009).

## 5 CONCLUSION

Comparison of cultivars in historic collections reveals shifts in plant phenotype in response to selective pressure for yield and agronomic adaptation. Our cultivar mixtures reinforced the role of plant–plant interactions underlying genetic yield gains. Plant and population perspectives are complementary. Reduced height—a plant trait—has consequences for allocation of dry matter *and* implications for yield mediated by reduced competitive ability. Darwin (1859) noted that in the most extreme environments, namely high latitude, high altitude or absolute deserts ‘the struggle for life is almost exclusively with the elements’; elsewhere, that is in all the environments that matter for agriculture, the relation of ‘organism to organism is the most important of all relations’. The contemporary definition of crop yield is a population trait, hence the crucial importance of plant–plant relations to understanding and improving crop yield.

## CONFLICT OF INTEREST

The authors declare no conflict of interest.

## AUTHOR CONTRIBUTION

C. Mariano Cossani: Conceptualization, Method, Investigation, Software, Formal analysis, Visualization, Writing—original draft. Victor O. Sadras: Conceptualization, Supervision, Method, Software, Formal analysis, Investigation, Visualization, Funding acquisition, Project administration, Writing ‐review and editing.

## Supporting information

Supplementary Material

Fig S1

Fig S2

## Data Availability

Data are available from authors upon request.
