# Supplementary material for: Symmetric response to competition in binary mixtures of cultivars associates with genetic gain in wheat yield
Source: Evol Appl. 2021 Jul 27;14(8):2064–78. doi: 10.1111/eva.13265 (PMC8372091; doi:10.1111/eva.13265)
Supplement: Supplementary file 1 — Supplementary Material [file EVA-14-2064-s002.docx]

**Supplementary Table 1:** Total inorganic nitrogen and plant available water in the soil profile (1 m) at sowing and topsoil (0.1 m) attributes in fields at two locations in South Australia. A week before sowing, soils were sampled to 1-m depth, separated in 0-0.1 m, 0.1-0.3 m, 0.3-0.6 m and 0.6-1 m intervals to measure water content gravimetrically, and inorganic nitrogen using a Skalar San^++^ Continuous Flow Analyser (Skalar Analytical B.V). Plant available water was calculated as the difference between actual water content and the lower limit for these soils retrieved from APSoil database <https://www.apsim.info/apsim-model/apsoil/>. APSoil number were CL028 for Roseworthy and 282 for Riverton.

| Soil attribute | Unit | Location |  |
| --- | --- | --- | --- |
|  |  | Riverton | Roseworthy |
| Total inorganic N | kg N-nitrate ha^-1^ | 106 | 125 |
| Plant available water | mm | 92 | 39 |
| pH_water_ | - | 5.5 | 8.1 |
| pH_CaCl2_ | - | 5.0 | 7.6 |
| Organic carbon | % | 1.9 | 1.9 |
| EC 1:5 | dS m^-1^ | 0.2 | 0.2 |
| Nitrate - N [1] | mg kg^-1^ | 39 | 28 |
| Ammonium - N [1] | mg kg^-1^ | 21.0 | <1 |
| [P [2]](file:///C:\UserData\Documents\2020\paper%20mixtures\mixtures\tabla%201.xlsx#RANGE!H43) | mg kg^-1^ | 59 | 65 |
| DGT-P | mg l^-1^ | 58 | 65 |
| K [2] | mg kg^-1^ | 380 | 370 |
| Ca [3] | mg kg^-1^ | 1480 | 5060 |
| Ca:Mg ratio | - | 3.3 | 7.8 |
| K:Mg ratio | - | 0.4 | 0.3 |
| B [4] | mg kg^-1^ | 1.1 | 2.4 |
| Fe [4] | mg kg^-1^ | 59 | 14 |
| Mn [4] | mg kg^-1^ | 35 | 5.6 |
| Cu [4] | mg kg^-1^ | 1.1 | 0.6 |
| Zn [4] | mg kg^-1^ | 2.4 | 4.9 |
| Dumas Total Nitrogen | % | 0.2 | 0.2 |
| [1] KCl extraction. |  |  |  |
| [2] Colwell. |  |  |  |
| [3] Ammonium acetate. |  |  |  |
| [4] Determined with EDTA extraction. | | |  |

**Supplementary Table 2.** ANOVA of wheat traits measured at maturity (grain yield, shoot biomass, harvest index, grain weight, grains per m^2^, plant height) and anthesis (δ^13^C, nitrogen nutrition index NNI, shoot biomass, percentage of water-soluble carbohydrates, and tiller fertility).


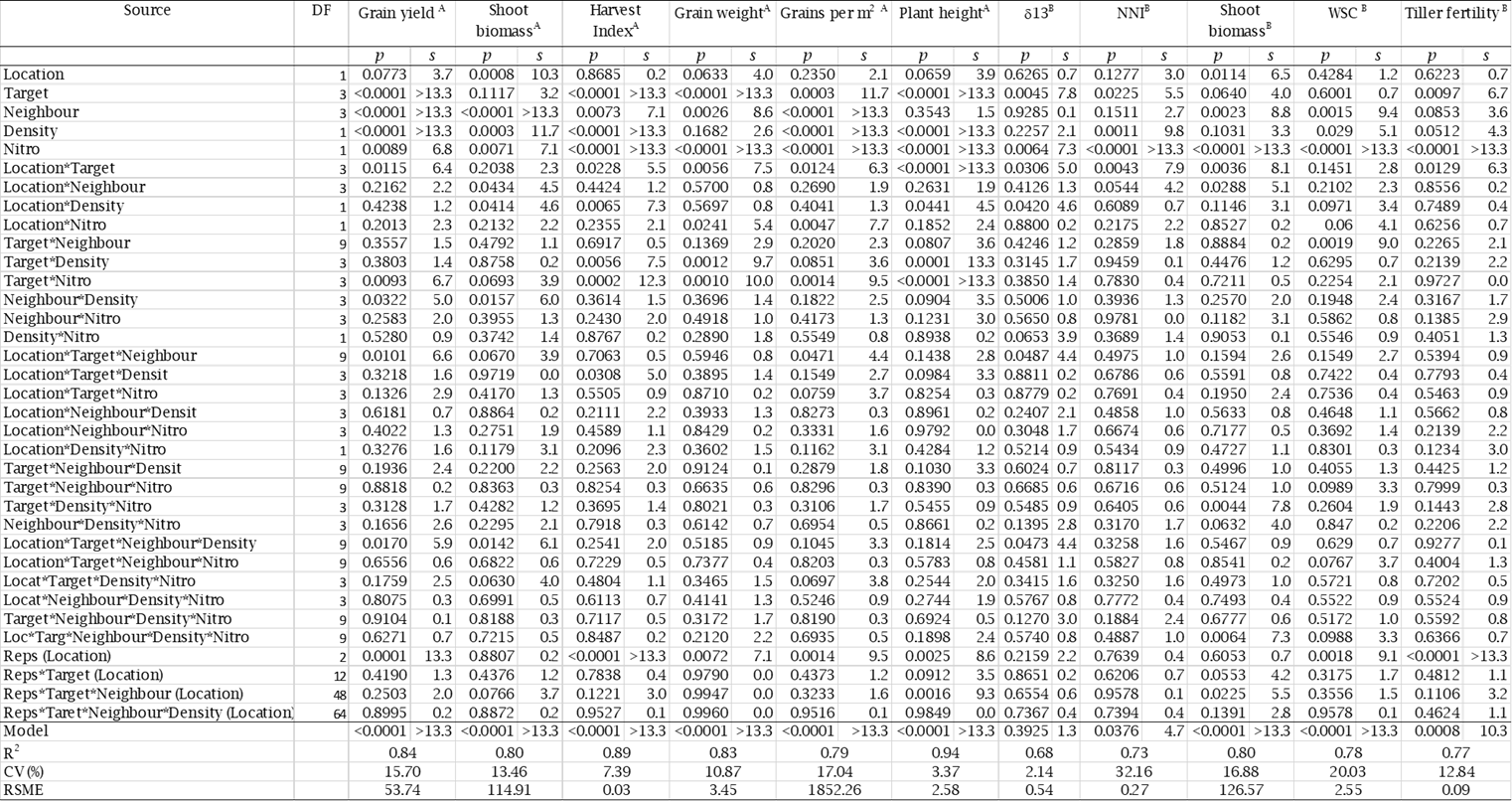
^A^  Measured at maturity. ^B^ Measured at anthesis.

**Supplementary Table 3.** Average (± standard error) grain yield and yield related traits in pure stands of four cultivars released between 1969 and 2015. Data are averaged across two locations, two stand densities and two nitrogen rates.

**Supplementary Figure 1.** Cumulative rainfall and evapotranspiration, and daily maximum (T_max_) and minimum (T_min_) temperature, and incident solar radiation during the growing season at Riverton (top row) and Roseworthy (bottom row), South Australia. CP indicates critical period for yield averaged across cultivars.

**
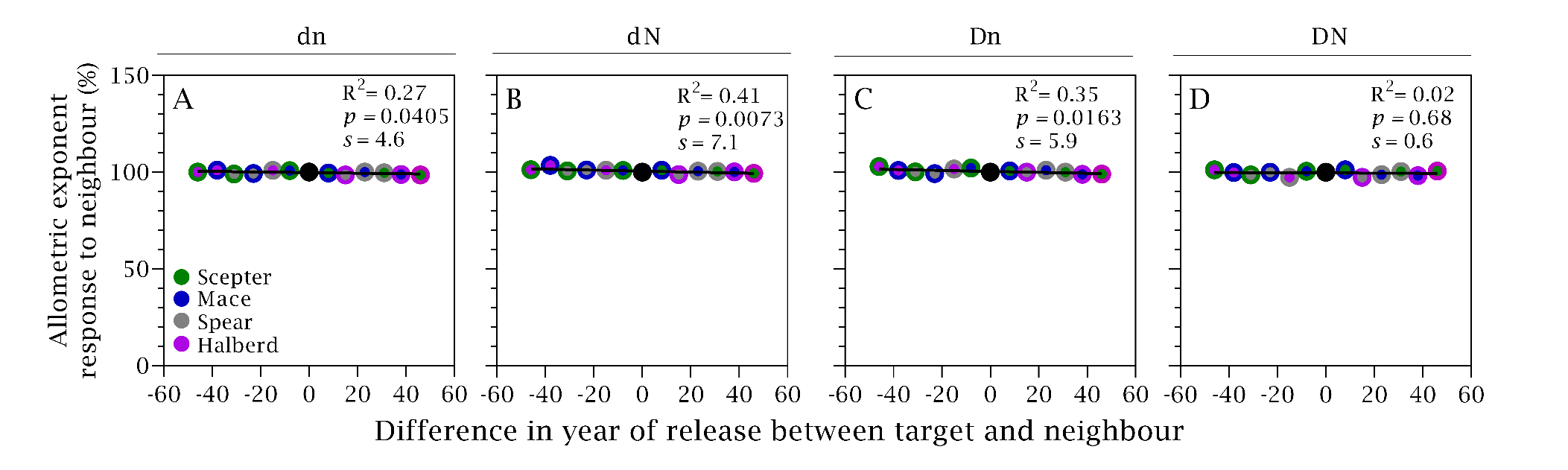
**

**Supplementary Figure 2.** Response to neighbour of allometric exponent as a function of the difference in year of release between target and neighbour for crops grown at two stand densities (d = 90 plants m^-2^ , D = 180 plants m^-2^), and two nitrogen rates (n = 0 kg ha^-1^, N = 100 kg ha^-1^), averaged for two locations. Allometric exponent is the slope of grain biomass vs rest-of-biomass at maturity in a log-log scale. Symbols: filling colour shows target and edge colour is neighbour, with black circle denoting pure stand. Solid lines are least squares regressions.
