# Supplementary figures and images for: Symmetric response to competition in binary mixtures of cultivars associates with genetic gain in wheat yield
Source: Evol Appl. 2021 Jul 27;14(8):2064–78. doi: 10.1111/eva.13265 (PMC8372091; doi:10.1111/eva.13265)

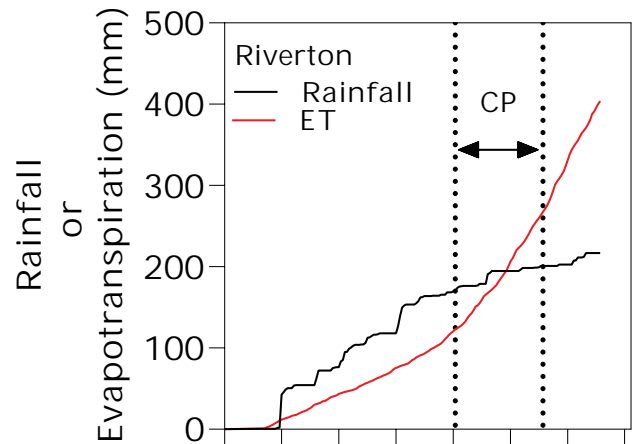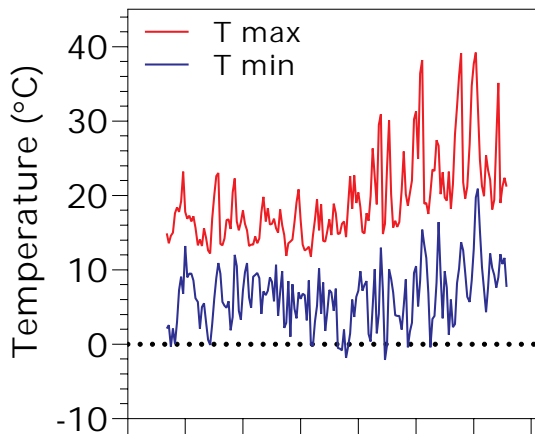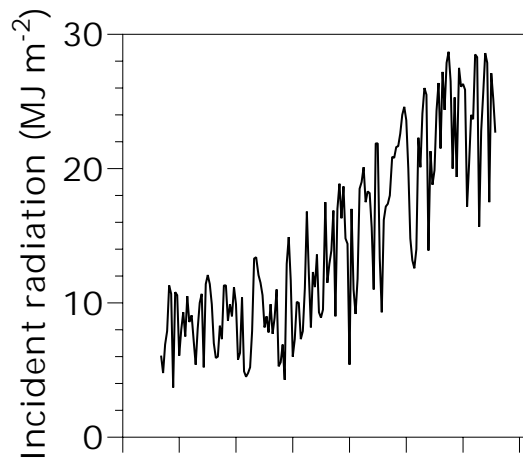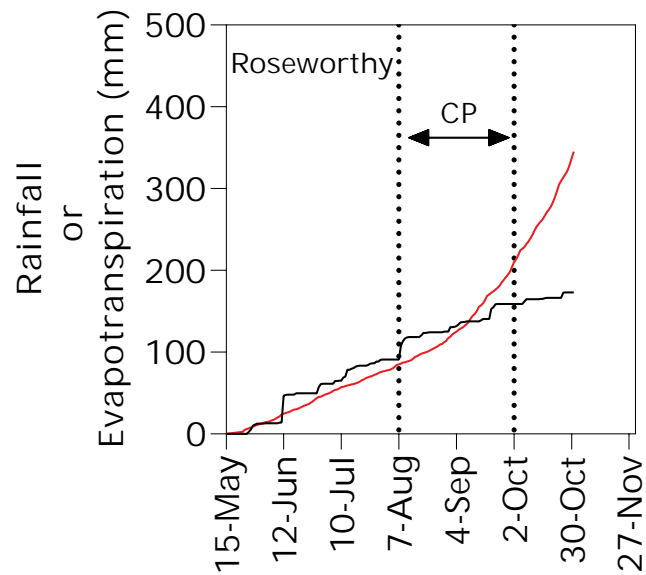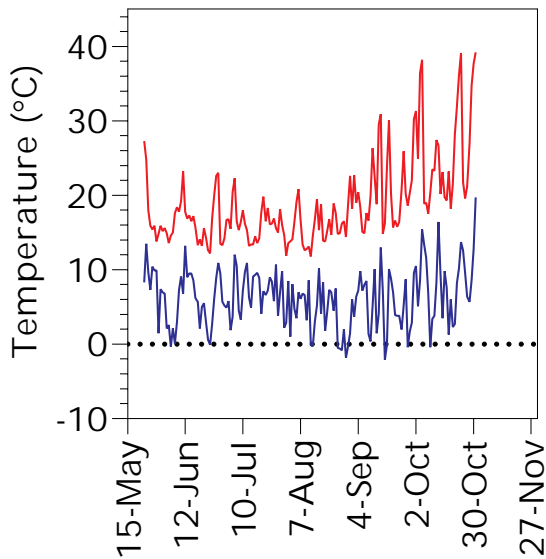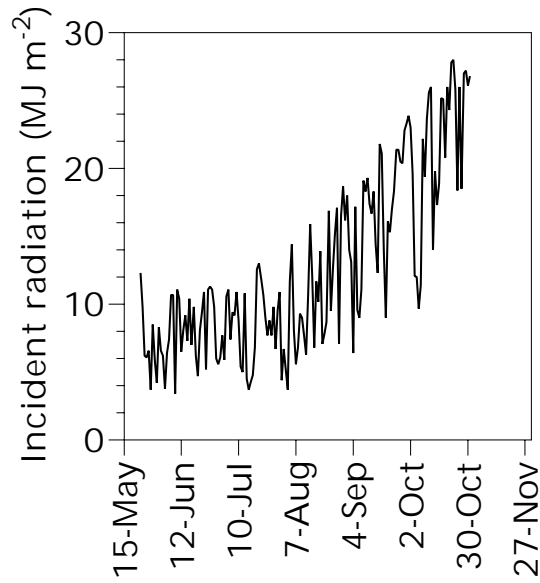

Date

Supplement: Supplementary file 2 — Fig S1 [file EVA-14-2064-s001.pdf]

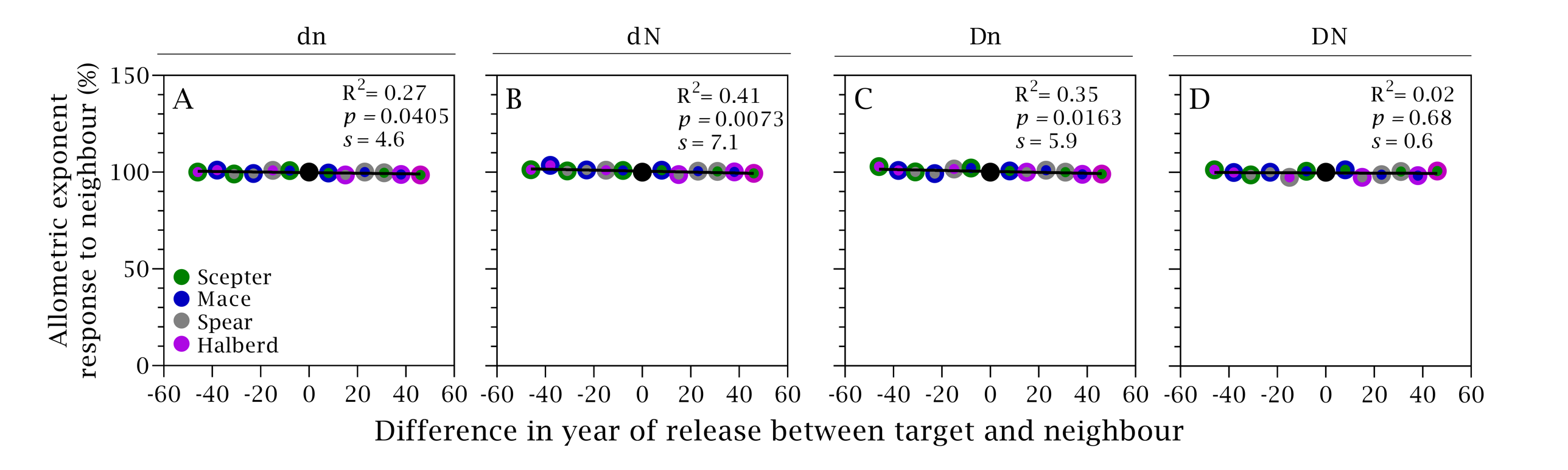

Supplement: Supplementary file 3 — Fig S2 [file EVA-14-2064-s003.tif]
